# Maltose binding protein-fusion enhances the bioactivity of truncated forms of pig myostatin propeptide produced in *E*. *coli*

**DOI:** 10.1371/journal.pone.0174956

**Published:** 2017-04-03

**Authors:** Sang Beum Lee, Sung Kwon Park, Yong Soo Kim

**Affiliations:** 1 Wide River Institute of Immunology, Seoul National University College of Medicine, Hongcheon, Gangwon-do, South Korea; 2 Department of Human Nutrition, Food and Animal Sciences, University of Hawaii at Manoa, Honolulu, HI, United States of America; 3 National Institute of Animal Science, RDA, Suwon, South Korea; Consiglio Nazionale delle Ricerche, ITALY

## Abstract

Myostatin (MSTN) is a potent negative regulator of skeletal muscle growth. MSTN propeptide (MSTNpro) inhibits MSTN binding to its receptor through complex formation with MSTN, implying that MSTNpro can be a useful agent to improve skeletal muscle growth in meat-producing animals. Four different truncated forms of pig MSTNpro containing N-terminal maltose binding protein (MBP) as a fusion partner were expressed in *E*. *coli*, and purified by the combination of affinity chromatography and gel filtration. The MSTN-inhibitory capacities of these proteins were examined in an *in vitro* gene reporter assay. A MBP-fused, truncated MSTNpro containing residues 42–175 (MBP-Pro42-175) exhibited the same MSTN-inhibitory potency as the full sequence MSTNpro. Truncated MSTNpro proteins containing either residues 42–115 (MBP-Pro42-115) or 42–98 (MBP-Pro42-98) also exhibited MSTN-inhibitory capacity even though the potencies were significantly lower than that of full sequence MSTNpro. In pull-down assays, MBP-Pro42-175, MBP-Pro42-115, and MBP-Pro42-98 demonstrated their binding to MSTN. MBP was removed from the truncated MSTNpro proteins by incubation with factor Xa to examine the potential role of MBP on MSTN-inhibitory capacity of those proteins. Removal of MBP from MBP-Pro42-175 and MBP-Pro42-98 resulted in 20-fold decrease in MSTN-inhibitory capacity of Pro42-175 and abolition of MSTN-inhibitory capacity of Pro42-98, indicating that MBP as fusion partner enhanced the MSTN-inhibitory capacity of those truncated MSTNpro proteins. In summary, this study shows that MBP is a very useful fusion partner in enhancing MSTN-inhibitory potency of truncated forms of MSTNpro proteins, and MBP-fused pig MSTNpro consisting of amino acid residues 42–175 is sufficient to maintain the full MSTN-inhibitory capacity.

## Introduction

Myostatin (MSTN) is a member of the transforming growth factor-β (TGF-β) superfamily and negatively regulates skeletal muscle growth and development with little effect on other tissues [1–3]. Similar to other TGF-β family member proteins, MSTN is translated as a precursor protein (preproMSTN) consisting of a signal sequence, an N-terminal propeptide domain (MSTNpro) and a C-terminal mature (active) domain [2, 4]. Upon entering into the endoplasmic reticulum, signal peptide is removed from preproMSTN, then proMSTN forms a disulfide-linked homodimer and is proteolytically processed at a conserved Arg-Arg-X-Arg site by the furin family of proprotein convertases to generate MSTNpro and mature MSTN [5, 6]. After the cleavage, two MSTNpros make a non-covalent complex formation with disulfide-linked homodimer of mature MSTN in a latent/inactive state to suppress MSTN binding to its receptor [6–8]. Administration or overexpression of MSTNpro has been shown to enhance skeletal muscle growth in laboratory animal species [4, 5, 9–14], supporting the inhibitory role of MSTNpro on MSTN activity.

Activation of MSTN from the latent state involves cleavage of MSTNpro by members of the bone morphogenetic proteins-1/tolloid (BMP-1/TLD) of metalloproteinases [4, 15]. The administration of wild type MSTNPro had no effect on skeletal muscle growth, while the administration of metalloproteinase-resistant mutant form of MSTNPro significantly enhanced skeletal muscle growth in mice [4], supporting the role of the metalloproteinase-cleavage site of MSTNpro on MSTN activity regulation.

According to a study [16], a region of human MSTNpro containing amino acid residues 42–115 was critical for MSTN inhibition. Similarly, a region of human MSTNpro containing residues 42–99 fully suppressed MSTN activity in co-transfection experiments [17], and a synthetic mouse MSTNpro peptide covering residues 45–68 was effective in suppressing MSTN activity [18]. We have also shown that maltose binding protein (MBP)-fused flatfish MSTN1pro region consisting of residues 45–100 had the same MSTN inhibitory potency as the MBP-fused full sequence flatfish MSTN1pro [19]. These studies together suggest that partial sequences of MSTNpro would be sufficient to maintain the full MSTN-inhibitory capacity.

In a previous study, we have successfully produced bioactive metalloproteinase-resistant mutant form of pig MSTNpro in *E*. *coli* [20] in an effort to examine the potential of MSTNpro to improve skeletal muscle growth of meat-producing animals. Since molecular size is a factor affecting the production yield in the *E*. *coli* expression system [21], the determination of MSTN-inhibitory domain of pig MSTMpro would contribute to enhancing the production yield of MSTNpro-type proteins having an equivalent potency to the full sequence MSTNpro. Thus, the objective of this study was to express and purify various truncated forms of pig MSTNpro to examine their MSTN-inhibitory capacities in an effort to investigate the region of pig MSTNpro critical for MSTN inhibition, as well as the minimum region possessing the full capacity for MSTN inhibition. Similar to previous studies, current results showed that inhibition of MSTN did not require full sequence of pig MSTNpro.It was also observed that MBP-fusion enhanced MSTN-inhibitory potency of truncated forms of MSTNpro proteins.

## Materials and methods

### Construction of expression vectors

**Fig 1:**
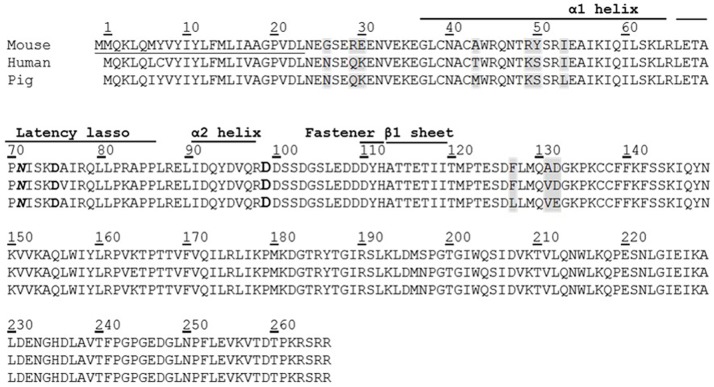
Amino acid sequence of pig MSTNpro aligned with those of mouse and human. Underline, bold italic characters (72), and bold characters (99) indicates signal sequence, N-Gly site (n-x-s/t), and putative proteolytic site by BMP-1/TLD proteases, respectively. The shaded amino acid residues sequence indicate different sequence identity among the three species. The domain names, including α1 helix, latency lasso, α2 helix, fastener, and β1 sheet, are based on a publication by Shi et al.[22].

Construction of plasmid containing full cDNA sequence of pig MSTNpro with mutation at the BMP-1/TLD proteolytic site (GenBank Accession No. KT932616) was reported previously [20], and this plasmid was used as a template in constructing four truncated forms of pig MSTNpro cDNAs. The four truncated forms of pig MSTNpro were named Pro42-218, Pro42-175, Pro42-115, and Pro42-98, following their starting and ending positions in the sequence. The DNA sequences of the above truncated forms of pig MSTNpro are found from GenBank Accession No. KT932617, KT932618, KT932619, and KT932620, respectively. The amino acid sequences of the truncated pig MSTNpro proteins can be found in Fig 1. For Gibson assembly cloning of the inserts into the XmnI site of *pMAL-c5x* expression vector (New England Biolab, MA, USA), PCR was performed using Q5 High-Fidelity DNA polymerase (New England Biolabs, MA, USA) with overlapping primer sets (Table 1). The PCR products were separated by agarose gel electrophoresis and purified using a commercial kit (Macherey-Nagel, Inc. Bethlehem, PA, USA) before use in DNA assembly reaction. Each Gibson assembly reaction (New England Biolabs, MA, USA) contained approximately 100 ng of insert and 50 ng of the XmnI-linearized *pMAL-c5x* vector, and incubated at 50°C for 30 min. After the assembly reaction, the reaction mixture was transformed into NEB 5α competent *E*. *coli* strain (New England Biolabs). After an overnight growth at 37°C, the *pMAL-c5x-Pro42-218*, *pMAL-c5x-Pro42-175*, *pMAL-c5x-Pro42-115*, and *pMAL-c5x-Pro42-98* plasmids were extracted to confirm correct insertion by colony PCR across the assembly junctions or by DNA sequence analysis. *E*. *coli* strain of K12TB1 (New England Biolab, MA, USA) was transformed with the expression constructs by the heat-shock method, and spread on Luria-Bertani (LB) (1.2% tryptone, 0.6% yeast extract and 0.8% NaCl, 0.2% glucose) agar plates containing 50 μg/mL ampicillin.

**Table 1:** Primer sequences used in Gibson assembly cloning.

Primer	Sequence
Pro42-218-F	gggatcgagggaaggAACGAAAACTCCGAACAAAAG
Pro42-218-R	catggacatatgtgaaatTTGCTTCAGCCAGTTCTG
Pro42-175-F	gggatcgagggaaggAACGAAAACTCCGAACAAAAGG
Pro42-175-R	catggacatatgtgaaatACGCAGGATCTGCACGAA
Pro42-115-F	gggatcgagggaaggTGTATGTGGCGTCAAAATAC
Pro42-115-R	catggacatatgtgaaatttaCGTGGTGGCATGGTAATC
Pro42-98-F	gggatcgagggaaggTGTATGTGGCGTCAAAATAC
Pro42-98-R	catggacatatgtgaaatttaACGTTGGACATCATACTG

Underlined, lower case letters indicate overlapping sequences with the pMAL-c5x expression vector.

### Expression and affinity purification of truncated forms of MBP-fused pig MSTNpro proteins

As described in detail previously [19], selected colonies were grown in 5 mL LB media containing 50 μg/mL ampicillin at 37^°^C overnight, and the 5 mL cultures were used to inoculate 250 mL LB media containing 50 μg/mL ampicillin. Protein expression was induced by adding IPTG to a final concentration of 0.3 mM when the cell density reached to an OD_600_ of 0.4 ~ 0.5, then the cultures were grown at 25^°^C for 15 hr. Cell pellets were collected, and soluble fractions of cell lysates were prepared, and maltose binding protein (MBP)-fused pig MSTNpro (MBP-MSTNpro) proteins were affinity-purified from soluble cell lysates using amylose resin as described previously [20]. After SDS-PAGE analysis of the presence of MBP-MSTNpro proteins in elution, fractions containing the target proteins were pooled. Protein concentration was determined by the Modified Lowry Protein Assay (Thermo Scientific, CA, USA) using BSA as a standard.

### Size-exclusion gel chromatography

The pooled, affinity-purified MBP-MSTNpro proteins were applied to a Superdex 75 column (Amersham-Pharmacia, NJ, USA) equilibrated with amylose affinity column buffer and eluted with the same buffer at a flow rate of 0.6 mL/min in a FPLC system. The absorbance was monitored at 280 nm, and 0.5 mL fractions were collected. The fractions were subjected to SDS-PAGE analysis to examine the purification.

### Examination of the binding of pig MBP-MSTNpro proteins to MSTN

The ability of pig MSTNpro proteins to bind to MSTN was examined using a pull-down assay as described in detail previously [19]. Three hundred ng of MSTN (R&D Systems, MN, USA) was added to either MBP-Pro42-175 (14.4 μg), MBP-Pro42-115 (12.0 μg), or MBP-Pro42-98 (12 μg), and mixed separately with 50 μL of amylose resin (New England Biolabs) to a final volume of 1 mL in a centrifugal spin column for 2 hr at room temperature. The concentrations of the above MBP-MSTNpro proteins were prepared to be 10-fold higher (480 nM) than that of MSTN (48 nM). The column was then centrifuged and washed with column buffer three times. The resin was then mixed with 20 μL of reducing SDS-PAGE loading buffer. After heating the sample at 100°C for 5 min, the loading buffer was carefully removed from the resin. The eluted proteins were then analyzed by SDS-PAGE with Coomassie blue staining. In another pull-down experiment, 75 ng of MSTN was added to various amounts of either MBP-Pro42-175 or MBP-Pro42-98, then followed by the same pull-down procedure. The concentration of MSTN was 6 nM, and the concentrations of MBP-Pro42-175 or MBP-Pro42-98 were 1, 15, 30, 45, and 60 nM. The eluted proteins from the pull-down experiment were fractionated by 15% SDS-PAGE, transferred to a PVDF membrane by electro-blotting, followed by immuno-blot analysis against MSTN.

### SDS-PAGE

SDS–PAGE was performed according to the method of Laemmli [23]. Samples were mixed with loading buffer in the presence or absence of 1.5% β-mercaptoethanol and boiled for 5 min prior to loading on the gel. Bands were stained with Coomassie Brilliant blue solution.

### Immuno-blot analysis

After blocking with Tris-buffered saline (20 mM Tris-HCl, 150 mM NaCl at pH 7.5) plus 0.01% Tween 20 (TTBS) for 2 hr at room temperature, the membranes were incubated with monoclonal anti-MSTN antibody [24] at a 1:1,000 dilution for 1 hr at room temperature. Membrane was washed (3 ×10 min) with TTBS and incubated with HRP-conjugated anti-mouse IgG (1:10,000, #7076, Cell Signaling, MA, USA) for 3 hr at room temperature. The membrane was washed (3 x 10 min), and HRP activity was detected using a chemiluminescence detection kit (GE Health Care, PA, USA), and visualized by exposing to X-ray film.

### pGL3-(CAGA)_12_-luciferase reporter assay

As described in detail previously [19], capacities of truncated, MSTNpro proteins to inhibit MSTN or GDF11 activities *in vitro* were measured using the pGL3-(CAGA)_12_-firefly luciferase reporter assay in HEK293 cells stably expressing (CAGA) _12_-luciferase gene construct [25]. Cells (40,000 cells/well) were seeded on a 96-well culture plate at in DMEM with 10% fetal calf serum, penicillin-streptomycin plus fungizone, and grown for 24 hr at 37°C with 5% CO_2_. 1 nM of MSTN (R&D Systems) or GDF11 (R&D Systems) plus various concentrations of commercial mouse MSTNpro (R&D Systems) or pig MSTNpro proteins in DMEM without serum were added to each well after removing the medium, and incubated for 24 hrLuminescence was measured using Veritas microplate luminometer (Turner Biosystems Inc., CA, USA) after adding Bright-Glo luminescence substrate (Promega, Madison, WI, USA). The % inhibition of MSTN activity was calculated by the following formula: % inhibition = (luminescence at 1 nM MSTN—luminescence at each ligand concentration)*100/(luminescence at 1 nM MSTN–luminescence at 0 nM MSTN). The MSTN-inhibitory activities were analyzed by regression analysis using Prism5 program (Graphpad, San Diego, CA). To examine the differences in MSTN-inhibitory capacity of these proteins and peptides, IC_50_ (ligand concentration inhibiting 50% of MSTN activity) values were estimated using a non-linear regression model defining dose response curve. The equation for the model was as follows: Y = Bottom + (Top–Bottom)/(1 + 10^(X–LogIC_50_)), where Y is % inhibition, Bottom is the lowest value of % inhibition, Top is the highest value of % inhibition, and X is Log ligand concentration. IC_50_ values were analyzed by ANOVA (Analysis of Variance) using the same program.

### Factor Xa digestion of MBP-Pro42-175 and MBP-Pro42-98

As described in detail previously [20], MBP-Pro42-175 or MBP-Pro42-98 were dialyzed against 100 vol of factor Xa reaction buffer (20 mM Tris, 100 mM NaCl, 2 mM CaCl_2_, pH 7.5) at 4°C overnight with 2 changes during dialysis. After dialysis, the proteins were incubated with factor Xa protease (New England BioLabs) for 24 hr at 4°C to remove MBP from these proteins. One ng of factor Xa was used to digest 50 ng of MBP-fusion proteins. The digestion was examined by SDS-PAGE. The factor Xa-digested proteins were then passed through the equilibrated amylose resin (New England BioLabs, MA) column to collect Pro42-175 and Pro42-98, followed by the removal of factor Xa with factor Xa Cleaning Kit (Novagen, San Diego, CA, USA) according to the protocol described by the manufacturer. After factor Xa digestion of MBP-Pro42-98, size-exclusion gel chromatography (Superdex 75, Amersham-Pharmacia) was used to purify Pro42-98 using a FPLC system.

### BMP-1 digestion of commercial MSTNpro

Five μg of commercial MSTNpro (R&D Systems) proteins in 35 μL of 25 mM HEPES buffer (pH 7.5) containing 0.01% (v/v) Brij-35 were mixed with 25 ng of human BMP-1 (R&D Systems) in 5 μL of the same buffer. After incubation for 1 hr at 37^°^C, digestion by BMP-1 was examined by SDS-PAGE.

## Results

### Expression and affinity purification of truncated, MBP-fused pig MSTNpro proteins

**Fig 2:**
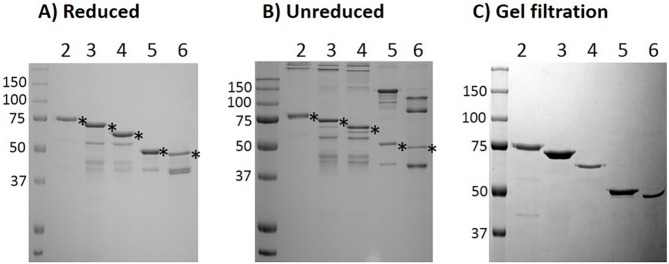
SDS-PAGE analysis of affinity-purified MBP-MSTNpro proteins. Soluble fractions of *E*. *coli* cell lysates from induced cultures were subjected to amylose-resin affinity column. Elution fractions were pooled and subjected to 12.5% SDS-PAGE gel, followed by Coomassie blue staining. Asterisks indicate affinity-purified MBP-MSTNpro proteins. Lanes 2, 3, 4, 5, and 6 indicate MBP-MSTNpro, MBP-Pro42-218, MBP-Pro42-175, MBP-Pro42-115, and MBP-Pro42-98, respectively.

Four MBP-fused, truncated forms of pig MSTNpro proteins were initially expressed, including MBP-Pro42-218, MBP-Pro42-175, MBP-Pro42-115, and MBP-Pro42-98. As was demonstrated with the full sequence of MBP-fused pig MSTNpro (MBP-MSTNpro) in our previous study [20], all of the four MBP-MSTNpro proteins were expressed in soluble forms in *E*. *coli* (data not shown). The result of SDS-PAGE of amylose affinity-purified proteins in reduced and non-reduced conditions are shown in Fig 2. The molecular masses of affinity-purified MBP-Pro42-218, MBP-Pro42-175, MBP-Pro42-115, and MBP-Pro42-98 were in agreement with the expected molecular masses of 63, 58, 51, and 49.5 kDa, respectively. The SDS-PAGE result in non-reduced condition showed a presence of some aggregations of the proteins (Fig 2B). Since there is a possibility that the aggregated proteins may have little bioactivity, the aggregates were removed by gel filtration (Fig 2C), and gel filtration-purified MBP-MSTNpro proteins were used in bioactivity examination. The yields of MBP-Pro42-218, MBP-Pro42-175, MBP-Pro42-115, and MBP-Pro42-98 after affinity purification were 8.0, 9.8, 32.2 and 38.0 mg/L culture, respectively (Table 2), indicating that the yield is in general inversely related to the molecular size.

**Table 2:** Yield of MBP-fused pig MSTNpro proteins recovered from amylose affinity purification.

	MBP-	MBP-	MBP-	MBP-
Steps	Pro42-218	Pro42-175	Pro42-115	Pro42-98
**Soluble Protein, mg/L**	110 ± 2.0	103 ± 5.6	129 ± 1.9	118 ± 3.0
**Affinity Purification, mg/L**	8.0^a^ ± 0.39	9.8^a^ ± 0.48	32.2^b^ ± 1.98	38.0^b^ ± 1.85

The truncated MBP-fused pig MSTNpro proteins were expressed at 25°C for 15 hr. Protein concentration was estimated by the Modified Lowry Protein Assay (Thermo Scientific, CA, USA) using BSA as a standard. Triplicates of 250 mL culture of each MSTNpro proteins were expressed to examine the recovery of proteins (mean ± SEM), and means were compared using Tukey’s significant difference test. The means not sharing the same superscript are different at P<0.05.

### Examination of MSTN- or GDF11-inhibitory capacity of MBP-fused pig MSTNpro proteins

**Fig 3:**
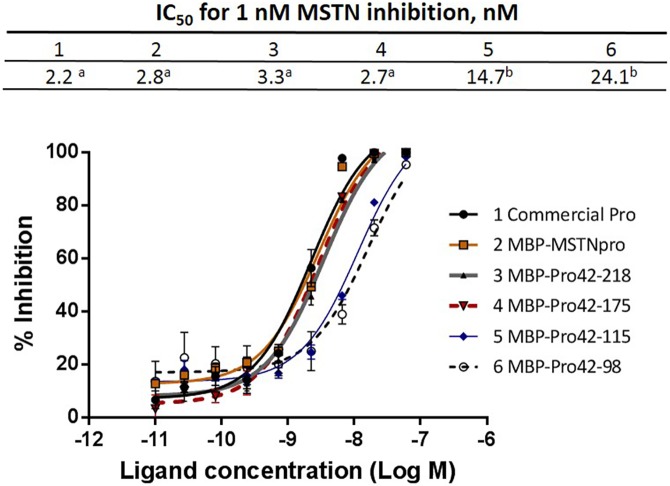
Inhibition of MSTN activity by MBP-pMSTNpro proteins. HEK293 cells stably expressing (CAGA) _12_-luciferase gene construct were seeded on a 96-well culture plate at 40,000 cells/well, and grown for 24 hr in DMEM with 10% fetal calf serum, antibiotic and antimycotic. Medium was removed, and MSTN (1 nM) and various concentrations (60–0 nM) of commercial MSTNpro (Commercial Pro) or MBP-MSTNpro proteins in DMEM were added to each well, followed by incubation for 24 hr. Medium was removed, and 60 μL of luminescence substrate were added and incubated for 3 minutes, followed by luminescence measurement. The % inhibition of MSTN activity was calculated by the following formula: % inhibition = (luminescence at 1 nM MSTN—luminescence at each ligand concentration)*100/(luminescence at 1 nM MSTN—luminescence at 0 nM MSTN). The error bars represent ± SEM (n = 6). The means of IC_50_ not sharing the same superscript are different at P<0.05.

All the MBP-MSTNpro proteins showed MSTN-inhibitory activity in the pGL3-(CAGA)_12_-luciferase reporter gene assay (Fig 3). The IC_50_ values of MBP-Pro42-218 (3.3 nM) and MBP-Pro42-175 (2.7 nM) to suppress 1 nM MSTN activity were not significantly different from either that of commercial MSTNpro (2.2 nM) or full sequence MBP-MSTNpro (2.8 nM), indicating that residues 42–175 is sufficient to maintain full MSTN-inhibitory potency. The IC_50_ values of both MBP-Pro42-115 (17.7 nM) and MBP-Pro42-98 (24.1 nM) to suppress 1 nM MSTN activity were significantly higher than those of commercial MSTNpro or full sequence MBP-MSTNpro, indicating lower potencies of MBP-Pro42-115 and MBP-Pro42-98 than the MBP-MSTNpro in suppressing MSTN activity.

**Fig 4:**
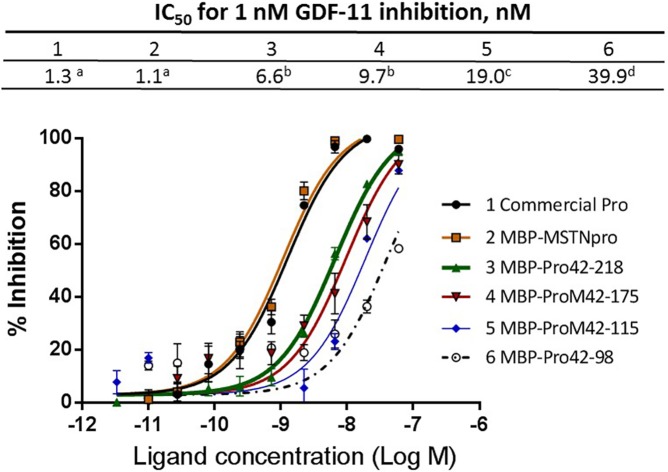
Inhibition of GDF-11 activity by MBP-pMSTNpro proteins. The assay procedure was the same as the MSTN inhibition assay (Fig 3) except that GDF-11 was used as a competing ligand instead of MSTN. Error bars represent ± SEM (n = 6). The means of IC_50_ not sharing the same superscript are different at P<0.05.

Since GDF11 is highly related to MSTN with 90% amino acid sequence homology [2, 26], the GDF11-inhibitory capacity of the pig MSTNpro proteins were examined (Fig 4). The IC_50_ values of full sequence MBP-MSTNpro to suppress 1 nM GDF11 (1.3 nM) was not different from that of commercial MSTNpro (1.2 nM). The IC_50_ values of MBP-Pro42-218 (6.6 nM) and MBP-Pro42-175 (9.7 nM) to suppress 1 nM GDF11 were not different from each other, but were significantly higher than that of full sequence MBP-MSTNpro (1.3 nM), indicating that unlike MSTN inhibition, MBP-Pro42-218 and MBP-Pro42-175 are not sufficient to maintain full GDF11-inhibitory potency. The IC_50_ value of MBP-Pro42-115 (19.0 nM) was significantly higher than those of MBP-Pro42-218 and MBP-Pro42-175, and the IC_50_ value of MBP-Pro42-98 (39.9 nM) was significantly higher than that of MBP-Pro42-115.

### Binding of MBP-Pro42-175, MBP-Pro42-115 or MBP-Pro42-98 to MSTN

**Fig 5:**
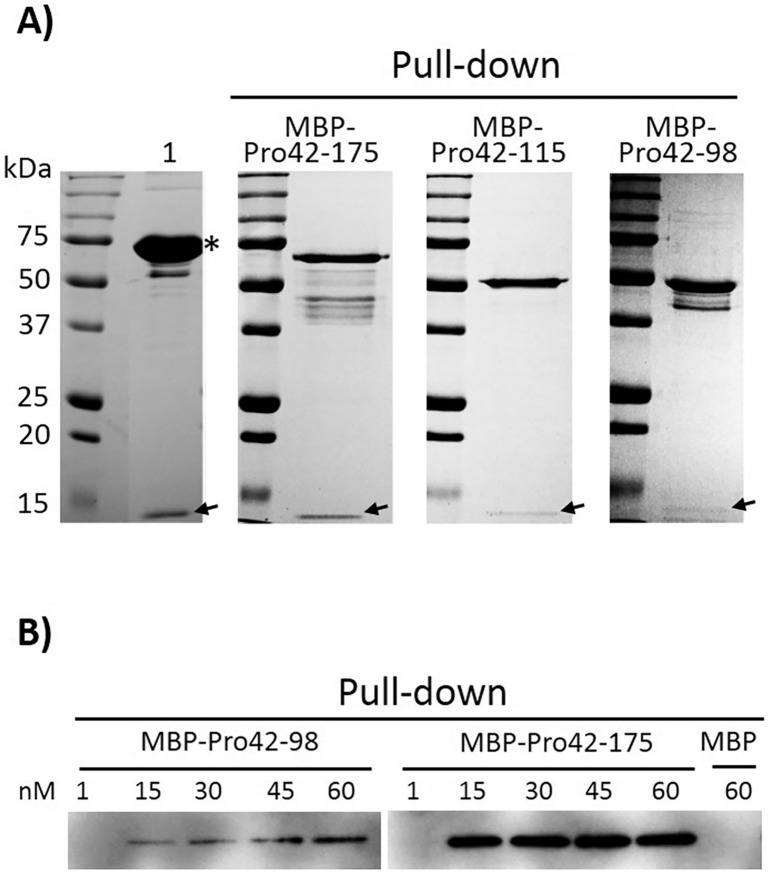
Binding of MBP-MSTNpro proteins to MSTN in a pull-down assay. **A)** Fifty microliters of amylose resin was mixed with either 480 nM of MBP-Pro42-175, MBP-Pro42-115, or MBP-Pro42-98 plus 48 nM of MSTN, separately to a final volume of 1 mL pull-down assay. The column was then washed with column buffer three times. Proteins bound to the amylose resin were then eluted with reducing SDS-PAGE loading buffer, and subjected to SDS-PAGE analysis. The commercial MSTN (lane 1) added in the pull-down experiment contained about 50-fold excess amount of BSA (*) as a carrier protein (R&D Systems). Arrows indicate MSTN bands. **B)** The same procedure describe in A was employed except that five different concentrations (1, 15, 30, 45, 60 nM) of either MBP-Pro42-175 or MBP-Pro42-98 with fixed concentration of MSTN (6 nM) were examined in the pull-down experiment. MBP (60 nM) binding to MSTN (6 nM) was also examined in the pull-down assay to serve as a negative control. Proteins bound to the amylose resin were eluted and subjected to immuno-blot analysis against monoclonal anti-MSTN antibody.

To examine whether the MSTN-inhibitory activities of MBP-MSTNpro proteins are associated with their bindings to MSTN, MSTN-binding of MBP-Pro42-175, MBP-Pro42-115, or MBP-Pro42-98 was examined in a pull-down assay. Since the MSTN-inhibitory capacity of MBP-Pro42-218 was not different from MBP-Pro42-175, the binding of MBP-Pro42-218 to MSTN was not examined. All three MBP-MSTNpro proteins showed their binding to MSTN (Fig 5A). The intensity of MSTN band from pull-down with MBP-Pro42-175 appeared to be stronger than those from pull-down with either MBP-Pro42-115 or MBP-Pro42-98, suggesting that MBP-Pro42-175 had greater binding affinity to MSTN than either MBP-Pro42-115 or MBP-Pro42-98. The BSA present in the commercial MSTN as a carrier protein (Fig 5A, lane 1) did not show any binding to the amylose column, indicating that the MSTN bands present in the pull-down assays were not due to a non-specific binding to the resin.

In order to confirm the difference in Coomassie blue-stained band intensity of MSTN in the pull-down assay was due to differences in binding affinities of MBP-Pro42-175, MBP-Pro42-115 or MBP-Pro42-98 to MSTN, another pull-down experiment was performed. Five different concentrations (1, 15, 30, 45, and 60 nM) of MBP-Pro42-175 or MBP-Pro42-98 were mixed with 6 nM of MSTN in this pull-down experiment, and MSTN band intensity was examined by Western blotting with ECL method (Fig 5B). As a negative control, MSTN binding to MBP was examined, and the result showed that MSTN did not bind to MBP at 60 nM. When 6 nM MSTN was mixed with 1 nM of either MBP-Pro42-175 or MBP-Pro42-98, no MSTN binding was detected. However, MSTN binding to MBP-Pro42-175 or MBP-Pro42-98 was detected when the concentrations of both proteins were beyond 15 nM. Greater amount of MSTN appeared to bind to MBP-Pro42-175 than to MBP-Pro42-98. With the increase of MBP-Pro42-98 from 15 nM to 60 nM in the pull-down reaction, the binding of MSTN to the protein appeared to increase gradually. In the MBP-Pro42-175 pull down assay, the amount of MSTN bound to MBP-Pro42-175 appeared not to significantly increase with the increase of its concentration from15 nM to 60 nM, suggesting a saturation of binding at 15 nM. This result, thus, indicates that the difference in MSTN-inhibitory capacities of the MBP-MSTNpro proteins was due to their differences in the binding affinity to MSTN.

### Removal of MBP from MBP-Pro42-175 or MBP-Pro42-98 by factor Xa digestion

A previous study reported that a GST-fused human MSTNpro consisting of residues 42–98 showed no MSTN-inhibitory capacity [16], suggesting that MBP potentially had a role in potentiating MSTN-inhibitory capacity of truncated forms of MSTN propeptides. MBP, thus, was removed from MBP-Pro42-98 by incubation with factor Xa to examine the role of MBP on MSTN-inhibitory capacity of MBP-Pro42-98. Since MBP-Pro42-175 had the full MSTN-inhibitory capacity, MBP was also removed from MBP-Pro42-175 by factor Xa digestion to examine whether MBP-fusion affected the MSTN inhibitory capacity of MBP-Pro42-175. The pMAL-c5x vector contained a factor Xa cleavage site between the fusion partner (MBP) and the target proteins.

**Fig 6:**
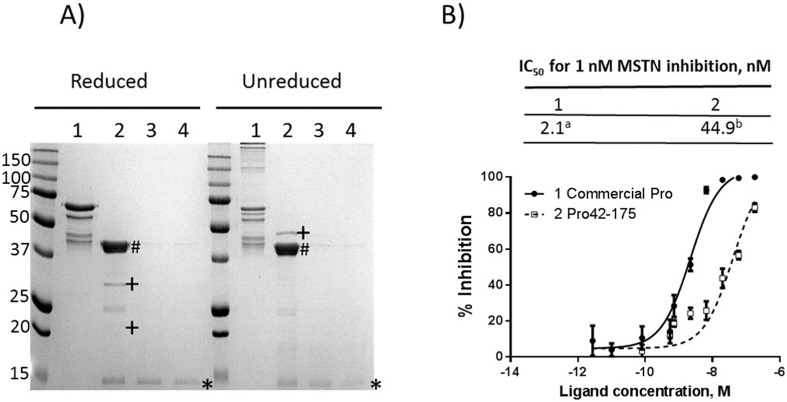
SDS-PAGE analysis of MBP-Pro42-175 after factor Xa cleavage and examination of MSTN-inhibitory capacity of Pro42-175. **(A)** After incubation of MBP-Pro42-175 with factor Xa, the supernatant was subjected to amylose affinity purification, followed by removal of factor Xa using a factor Xa affinity column. Protein samples collected during the cleavage and purification steps were subjected to 15% SDS-PAGE analysis, and visualized with Coomassie blue staining. Lane 1, MBP-Pro42-175; lane 2, MBP-Pro42-175 digested with factor Xa; lane 3, Pro42-175 purified by amylose affinity; lane 4, Pro42-175 with factor Xa being removed; #, MBP; +, factor Xa (disulfide linked heterodimer under non-reduced condition); *, Pro42-175. **(B)** MSTN-inhibitory capacity of purified Pro42-175 was measured using the (CAGA) _12_-luciferase reporter gene assay. The means of IC_50_ not sharing the same superscript are different at P<0.05.

The digestion of MBP-Pro42-175 led to appearance of two major bands of around 43 kDa and 15 kDa (Fig 6A), indicating a cleavage of MBP-Pro42-175 (53 kDa) into MBP (43 kDa) and Pro42-175 (15 kDa). The Pro42-175 was purified by removal of MBP and factor Xa (Fig 5A). Like the native MSTNpro, the Pro42-175 did not form homo dimer in unreduced condition (Fig 4A). When the MSTN-inhibitory capacity of the Pro42-175 was examined (Fig 6B), the IC_50_ value of Pro42-175 (44.9 nM) to suppress 1 nM MSTN was about 15 fold higher than that of MBP-Pro42-175 (2.7 nM), indicating that the presence of MBP as a fusion partner helped the binding of MBP-Pro42-175 to MSTN, leading to enhanced MSTN-inhibitory capacity of MBP-Pro42-175.

**Fig 7:**
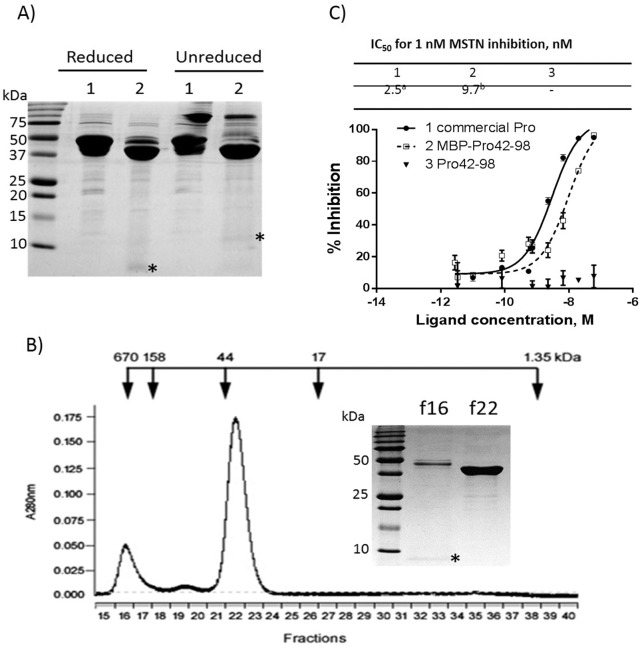
SDS-PAGE analysis of MBP-Pro42-98 after factor Xa cleavage and examination of MSTN-inhibitory capacity of Pro42-98. **(A)** After incubation of MBP-Pro42-98 with factor Xa, the total reaction mixture was centrifuged at 10,000 g for 3 min. The supernatant (soluble fraction) was subjected to 15% SDS-PAGE analysis under reduced and unreduced conditions, then visualized with Coomassie blue. *, Pro42-98. **(B)** The supernatant was subjected to gel filtration, and fractions 16 (f16) and 22 (f22) were subjected to SDS-PAGE analysis under reduced condition. *, Pro42-98. **(C)** MSTN-inhibitory capacity of purified Pro42-98 (f16) was measured using the (CAGA) _12_-luciferase reporter gene assay. The means of IC_50_ not sharing the same superscript are different at P<0.05.

The MBP-Pro42-98 was also incubated with factor X, and the digestion led to the appearance of two bands around 43 kDa and 7 kDa under reduced condition (Fig 7A), indicating a cleavage of MBP-Pro42-98 (49.5 kDa) into MBP (43 kDa) and Pro42-98 (6.5 kDa). Unlike the commercial MSTNpro or Pro42-175, the Pro42-98 formed a homo-dimer under an unreduced condition (Fig 7A). When the digested reaction mixture was applied to an amylose affinity column, unlike the Pro42-175, the Pro42-98 did not appear in pass-through faction, but appeared in the elution fractions (data now shown), suggesting a potential complex formation with MBP or other undigested MBP-Pro42-98. We, thus, applied the digested reaction mixture to a size exclusion chromatography column. Examination of fractions containing elution peaks revealed that the Pro42-98 (6.9 kDa) appears in a void volume together with undigested MBP-Pro42-98 while the MBP (43 kDa) appears in its expected elution fraction (Fig 7B), indicating a soluble aggregate formation together with undigested MBP-Pro42-98. We examined the MSTN-inhibitory capacity of the fraction (f16) containing the Pro42-98, and detected little MSTN-inhibitory capacity (Fig 7C).

### BMP-1 digestion of commercial MSTNpro

**Fig 8:**
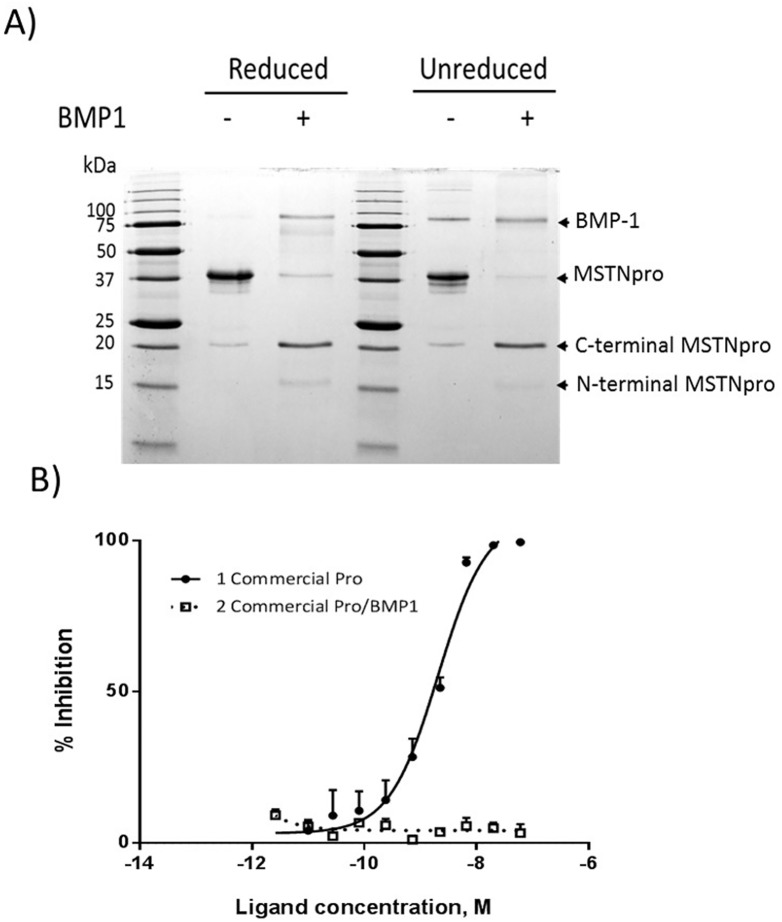
SDS-PAGE analysis of commercial MSTNpro after BMP-1 digestion and examination of MSTN-inhibitory capacity of BMP-1 digested commercial MSTNpro. **(A)** After incubation of commercial MSTN-pro with BMP-1, the total reaction mixture was centrifuged at 10,000 g for 3 min. The supernatant (soluble fraction) was subjected to 15% SDS-PAGE analysis under reduced and unreduced conditions, then visualized with Coomassie blue. **(B)** MSTN-inhibitory capacity of BMP-1 digested commercial MSTN-pro was measured using the (CAGA) _12_-luciferase reporter gene assay.

BMP-1 protease cleaves MSTNpro between Arg-98 and Asp-99 [4, 15, 20]. Since Pro42-98 formed a homo-dimer in unreduced condition (Fig 7A), we examined whether the N-terminal fragment (residues 23–98 in Fig 1) cleaved from commercial MSTNpro by incubation with BMP-1 forms a homo-dimer under unreduced condition. Under the incubation with BMP-1, the commercial MSTNpro produced two fragments with molecular masses of about 20 kDa and 16 kDa (Fig 8A). The expected molecular mass of C-terminal fragment (residues 99–266 in Fig 1) is 19 kDa, suggesting that the 20 kDa band is the C-terminal fragment of cleaved MSTNpro, and the 16 kDa band is the glycosylated N-terminal fragment (residues 23–98 in Fig 1). Unlike the Pro42-98, the N-terminal fragment did not form homo-dimer under unreduced condition (Fig 8A). It was suspected whether the residues 23–41 has an influence on the disulfide bond formation via Cys-42. We, thus, expressed and purified MBP-Pro23-98, then removed MBP by factor Xa cleavage (S1 Fig). Like the Pro42-98, Pro23-98 formed a homodimer (S1 Fig), indicating that the residues 23–41 does not prevent the disulfide bond formation via Cys-42. It is, thus, speculated that the glycosylation at Asn-70 is probably involved in the prevention of disulfide bond formation via Cys-42 in native BMP-1 digested N-terminal MSTNpro fragment. When we examined the MSTN-inhibitory capacity of BMP-1 digested commercial MSTNpro, no MSTN-inhibitory activity was found (Fig 8C). The MBP-removed Pro24-98 also showed no MSTN-inhibitory capacity (S1 Fig).

## Discussion

The current study shows that a MBP-fused pig MSTNpro consisting of amino acid residues 42–175 is sufficient to maintain the full MSTN-inhibitory capacity. Our recent study also demonstrated that MBP-fused flatfish MSTN1pro domain consisting of residues 45–100 had a full MSTN-inhibitory capacity [19]. Similarly, it was reported that a GST-fused human MSTNpro consisting of amino acid residues 42–218 had the full MSTN-inhibitory capacity [16]. These studies together indicate that full sequence of MSTNpro may not be required for a full MSTN-inhibitory capacity. It has, however, been unknown whether a fusion partner could play a role in modulating MSTN-inhibitory capacity of the truncated forms of MSTNpro, and the current results show that MBP fusion enhances MSTN-inhibitory capacity of the truncated forms of MSTNpro. The MSTN-inhibitory capacity of MBP-removed MSTNpro domain containing residues 42–175 was almost 20-fold lower as compared to MBP-fused MSTNPro domain containing residues 42–175, and MBP-removed MSTNPro domain containing residues 42–98 showed little MSTN-inhibitory capacity. Of note, our current and previous results [20, 27] showed that MBP alone did not bind MSTN to suppress its activity. Notably, a GST-fused human MSTNpro consisting of residues 42–98 showed no MSTN-inhibitory capacity [16], while we observed MSTN-inhibitory capacity of MBP-fused MSTNPro domain containing residues 42–98, indicating that the enhancement of MSTN-inhibitory capacity is more likely to be confined to MBP rather than to other fusion partners. Even though it is well known that MBP is an effective solubility enhancer as a fusion partner [28, 29], as far as we know, this is the first demonstration of biological activity enhancement of a passenger protein by MBP fusion. With regard to the mechanism of solubility enhancement by MBP, it has been proposed that MBP transiently binds folding intermediates or aggregation-prone passenger protein, keeping the passenger protein in a soluble state until either spontaneous or chaperon-mediated folding can occur [29]. The mechanisms of how MBP enhances the MSTN-inhibitory capacity of truncated forms of pig MSTNpro is not known currently. Based on the proposed mechanism of solubility enhancement by MBP, it is, however, speculated that MBP fusion possibly stabilized the folding state of the Pro42-98, keeping Pro42-98 from the formation of homogeneous or heterogeneous soluble aggregates. In fact, our data (Fig 7) indicated the heterogeneous soluble aggregate formation upon removal of MBP. To clearly explain the mechanisms of how MBP enhances the MSTN-inhibitory capacity of truncated forms of pig MSTNpro, future studies, such as molecular modeling or determination of 3D structure, are warranted.

The cleavage of MSTNpro between Arg-98 and Asp-99 by the bone morphogenetic protein-1/tolloid (BMP-1/TLD) family of metalloproteinases and subsequent release of MSTN from the latent complex is known to be the mechanism for MSTN activation *in vivo* [4, 15], implying that MSTNpro domains containing amino acid residues below Asp99 has little MSTN-inhibitory capacity. The result of Jiang et al.[16] in which a GST-fused human MSTNpro consisting of residues 42–98 showed no MSTN-inhibitory capacity supports the mechanism, as well as our current result. On the other hand, it has been reported that synthetic MSTNpro peptides containing residues in the N-terminal fragment (below Arg-98) of BMP-1-digested MSTNpro have MSTN-inhibitory capacity in vitro [17–19], as well as enhancement of muscle growth upon administration of the peptides[17, 18]. The apparent contradictory results can be related to the administration dose of peptides. Studies on mutational analysis and determination of X-ray crystallographic structure of proTGF-β1 [22, 30] indicate that the BMP-1-digested N-terminal fragment of MSTNpro, containing the α1 helix, latency lasso loop and α2 helix (Fig 1), blocks receptor binding of MSTN, thus play an essential role for MSTN binding and inhibition of MSTN activity. However, the MSTN-binding affinity of the BMP-1-digested N-terminal fragment of MSTNpro appears to be very weak, it is thus speculated that C-terminal region from the BMP-1-digested point stabilizes the binding to MSTN. In support of this speculation, the MSTNpro peptides containing residues in the BMP-1-digested N-terminal fragment required micromolar concentration to suppress MSTN activity [17–19].

In summary, this study shows that MBP-fusion enhances the MSTN-inhibitory capacity of truncated forms of pig MSTNpro, and MBP-fused pig MSTNpro consisting of amino acid residues 42–175 is sufficient to maintain the full MSTN-inhibitory capacity.

## Supporting information

S1 Fig SDS-PAGE analysis of MBP-Pro24-98 proteins after factor Xa cleavage. **(A)** After incubation of MBP-Pro24-98 with factor Xa, the total reaction mixture was centrifuged at 10,000 g for 3 min to soluble and in soluble fractions. Soluble fractions were subjected to 15% SDS-PAGE analysis under reduced (lane 1) and unreduced (lane 2) conditions, then visualized with Coomassie blue. *, Pro24-98. **(B)** The supernatant was subjected to gel filtration, and fractions 16, 19, and 22 were subjected to SDS-PAGE analysis under reduced condition. Arrow head indicate Pro24-98. **(C)** MSTN-inhibitory capacity of purified Pro24-98 (f16) was measured using the (CAGA) _12_-luciferase reporter gene assay. The means of IC_50_ not sharing the same superscript are different at P<0.05.
(JPG)
