# Supplementary figures and images for: Maltose binding protein-fusion enhances the bioactivity of truncated forms of pig myostatin propeptide produced in E. coli
Source: PLoS One. 2017 Apr 3;12(4):e0174956. doi: 10.1371/journal.pone.0174956 (PMC5378391; doi:10.1371/journal.pone.0174956)

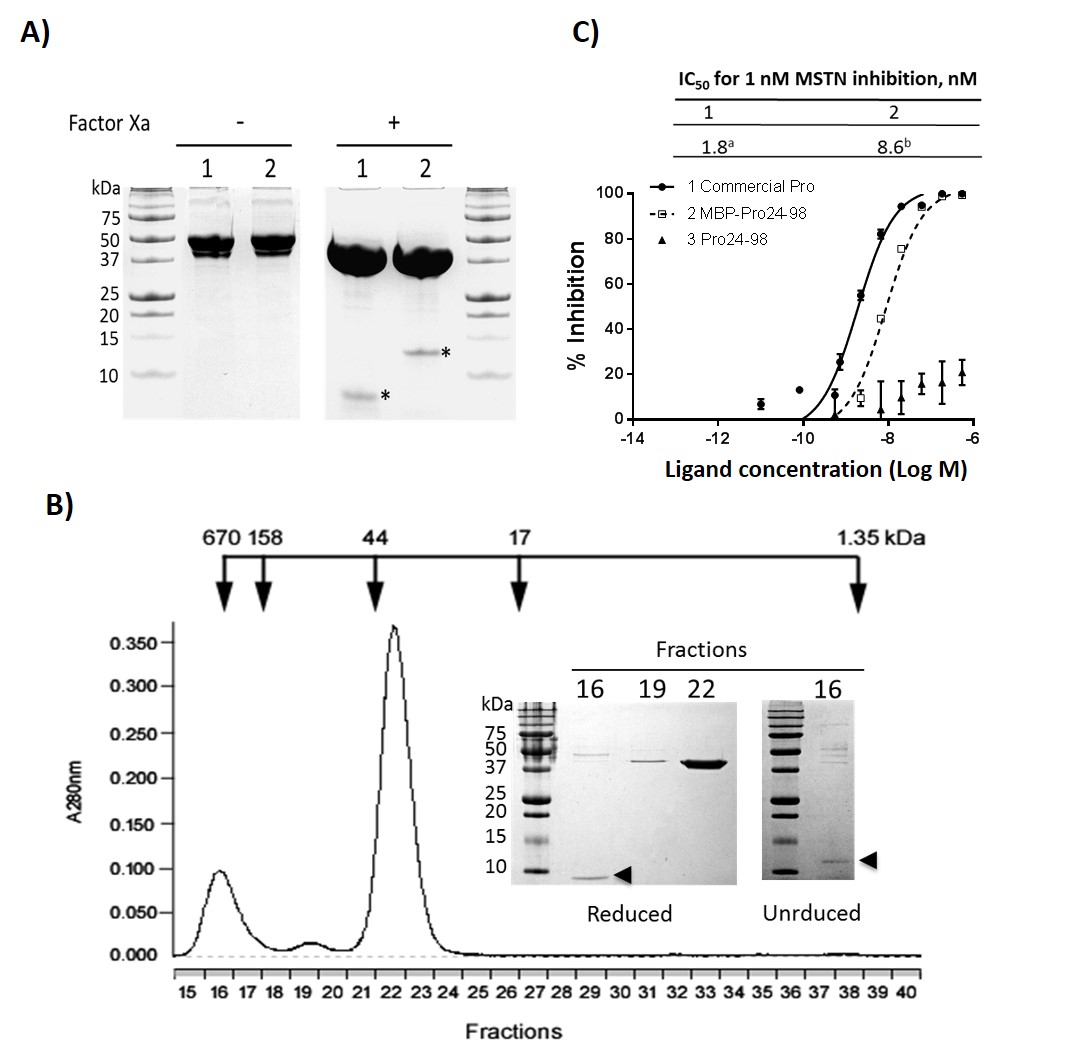

Supplement: S1 Fig — (A) After incubation of MBP-Pro24-98 with factor Xa, the total reaction mixture was centrifuged at 10,000 g for 3 min to soluble and in soluble fractions. Soluble fractions were subjected to 15% SDS-PAGE analysis under reduced (lane 1) and unreduced (lane 2) conditions, then visualized with Coomassie blue. *, Pro24-98. (B) The supernatant was subjected to gel filtration, and fractions 16, 19, and 22 were subjected to SDS-PAGE analysis under reduced condition. Arrow head indicate Pro24-98. (C) MSTN-inhibitory capacity of purified Pro24-98 (f16) was measured using the (CAGA) 12-luciferase reporter gene assay. The means of IC50 not sharing the same superscript are different at P<0.05. (JPG) [file pone.0174956.s001.jpg]
